# Reconstructing social mixing patterns via weighted contact matrices from online and representative surveys

**DOI:** 10.1038/s41598-022-07488-7

**Published:** 2022-03-18

**Authors:** Júlia Koltai, Orsolya Vásárhelyi, Gergely Röst, Márton Karsai

**Affiliations:** 1 Computational Social Science and Research Center for Educational and Network Studies, Centre for Social Sciences, Budapest, 1097 Hungary; 2 Faculty of Social Sciences, Eötvös Loránd University, Budapest, 1117 Hungary; 3 Department of Network and Data Science, Central European University, 1100 Vienna, Austria; 4 Laboratory for Networks, Technology and Innovation, Centre for Advanced Studies, Budapest Corvinus University, Budapest, 1093 Hungary; 5 Centre for Interdisciplinary Methodologies, University of Warwick, Coventry, United Kingdom; 6 Bolyai Institute, University of Szeged, Szeged, 6720 Hungary; 7 Alfréd Rényi Institute of Mathematics, Budapest, 1053 Hungary

**Keywords:** Scientific data, Statistics, Applied mathematics

## Abstract

The unprecedented behavioural responses of societies have been evidently shaping the COVID-19 pandemic, yet it is a significant challenge to accurately monitor the continuously changing social mixing patterns in real-time. Contact matrices, usually stratified by age, summarise interaction motifs efficiently, but their collection relies on conventional representative survey techniques, which are expensive and slow to obtain. Here we report a data collection effort involving over $2.3\%$ of the Hungarian population to simultaneously record contact matrices through a longitudinal online and sequence of representative phone surveys. To correct non-representative biases characterising the online data, by using census data and the representative samples we develop a reconstruction method to provide a scalable, cheap, and flexible way to dynamically obtain closer-to-representative contact matrices. Our results demonstrate that although some conventional socio-demographic characters correlate significantly with the change of contact numbers, the strongest predictors can be collected only via surveys techniques and combined with census data for the best reconstruction performance. We demonstrate the potential of combined online-offline data collections to understand the changing behavioural responses determining the future evolution of the outbreak, and to inform epidemic models with crucial data.

## Introduction

The spread of directly transmitted diseases such as COVID-19 is largely driven by social interactions and mixing patterns of people^1–3^. While person-to-person transmission typically occurs in close contacts^4,5^, local transportation, commuting, or global travels allow the disease to reach distant territories. Mobility patterns of entire populations can be traced from data coming from transportation or personal digital devices^6^, yet the observation of social interactions is still not obvious. The estimation of interactions and mixing patterns via social proximity^7^, commonly coded as contact matrices^1,8,9^, is difficult, especially when we can only observe a fraction of the population. Furthermore, most newly developed contact tracing apps only notify users if they are in the proximity of an infected person, and due to privacy concerns^10^ they cannot collect personal information about app users or their devices, and cannot share data with third party users^11^. Therefore these solutions cannot provide representative contact pattern data for a larger population. By combining anonymous online data collection techniques with conventional, representative sample based survey methods, we propose a privacy protecting, dynamic, economical, scalable and efficient solution to this problem. Our newly developed large-scale online data collection method, similarly to any other method based on voluntary participation, suffers from unrepresentativity. To overcome this limitation, we suggest a weighting methodology for the large-scale online data, using a smaller-scale representative sample simultaneously. This methodology solves the puzzle how voluntary online questionnaires may produce more valid and dynamic contact matrices to inform epidemic models.

The simplest approach to model an epidemic assumes that contacts between any two individuals occur randomly with equal probability. This so called *homogeneous mixing* assumption dominated the early years of mathematical and computational epidemiology and lead to the seminal results on the dynamics of infectious diseases^12^. However, the heterogeneity of populations called for more refined assumptions to bring the models closer to reality. One successful direction assumes *networked populations* where the social interaction structure of people is taken as the underlying skeleton for epidemic transmission^13^. Social networks commonly appear with various structural heterogeneity^14^, which crucially amplify the chances of global spreading scenarios^13^ while making them easier to immunise^15^ in case their global structure is known. However, collecting data about the precise social network of a large population is difficult. Thus, a middle way approach between homogeneously mixed and networked populations is necessary, which is proposed by *contact matrices* representing the aggregated probabilities that different groups of people are in contact with each other^1,8,9^. Most commonly, contacts between age groups are considered, but family structure, gender, education, and other socio-demographic variables have also been used for such stratification^16–18^. The advantages of contact matrices are manifold, as they can be easily integrated to conventional mathematical frameworks to describe the dynamics of an epidemic. Further, they are privacy preserving as they only record aggregated information, yet effectively breaking the homogeneous mixing assumption within a population. They can be dynamically collected and re-scaled to simulate the effects of social distancing or the isolation of different groups for scenario testing of epidemic outcomes.

International and national efforts were implemented worldwide to estimate locally relevant contact matrices for epidemic modelling. One of the largest and earliest effort was carried out by Mossong *et al.* in the POLYMOD project^1^, where in eight European countries 7,290 participants were asked to provide their daily contact data to estimate the aggregated age contact matrices. Following these efforts similar studies^19^ have been conducted in various other countries around the world^18,20–32^, while several contact matrix estimation methods were also developed^8,33^. One important study was published by Prem *et al.*^9^, who, based on the POLYMOD results and local census data, estimated the contact matrices of 152 countries by using Markov Chain Monte Carlo simulation. All these studies were established on a few paradigms of data collection methods^34–36^. Several questionnaire based data collection campaigns were carried out using CATI, CAWI or CAPI survey methodologies^18,30,31^. They commonly collected easily interpretable data, sometimes from representative samples using careful sampling design. Such data collection efforts became crucially relevant lately due to the COVID-19 pandemic, which called for contact matrix data collection campaigns in many countries^21,23,37,38^. Nevertheless, most of these data suffers from limited sample size, high cost of data collection, and, except some recent examples^23,37^, as they were cross-sectional studies, they completely missed to capture any dynamical change of contact patterns during normal or pandemic periods. On the other hand, online questionnaires and behavioural data collection apps may open new ways to solve these problems. They can reach large populations up to millions of people, while collecting data dynamically, even with changing content, for relatively small costs. However, they may press on privacy issues and due to the voluntary participation, they fall short on providing a representative sample of the observed population. The later crucially limits their direct applicability; as any interpretation drawn from their results need to be handled with caution. Thus, the question remains, how can one exploit all the advantages what online data collection methods provide, while ensuring the privacy of the respondents and the representativeness of the data collected?

### Actual circumstances

The recent COVID-19 pandemic called for an immediate answer to this question. In the early days of March 2020, as the COVID-19 pandemic started to unfold in Hungary, scientists from diverse fields were requested to develop country specific epidemic models. This effort was supported by a never seen initiative, in which mobile phone providers and health authorities shared their data to help realistic data-driven modelling approaches. However, one important data was missing from the very beginning: the spatially and demographically detailed mixing patterns of the population’s different age groups. Although estimated^9^ contact matrices were available for Hungary from earlier periods, the actual challenge was to continuously monitor the changes in contact patterns and to measure the societal responses - like social distancing or self-protection - to the COVID-19 related nationwide regulations. The Hungarian Data Provider Questionnaire (“Magyar Adatszolgáltató Kérdőív” - MASZK)^39^ was developed for these purposes. The voluntary and anonymous online survey (designed by scientists and software engineers^40^), is part of a larger project aiming to observe and model the unfolding COVID-19 pandemic in Hungary^41,42^. Beyond collecting static information about the respondents’ demography, domicile, education level, or family structure, the primary goal of the questionnaire was to dynamically monitor the daily changes in the contact pattern of people in order to calculate the age contact matrices in real time. Additionally, dynamic data was collected about the respondents’ employment status, working conditions, physical and mental well-being, and their compliance with recommended or mandatory self-protection measures during the months of emergency state and beyond. This rolling anonymous online data collection campaign is ongoing up to date (Summer 2021) and reached over $2.3\%$ of the population in Hungary recording over 480, 000 questionnaires from more than 232, 000 individuals, mounting up to the largest data ever collected for this purpose, to our knowledge.

### Problem and focus

However, as participation was voluntary, just as any data collected in similar ways, the obtained dataset was not representative for the population of Hungary. To estimate the level and dimensions of unrepresentativity, and to have generalizable results, we performed parallel data collection campaigns based on the same questionnaire, but conducted on a smaller representative sample of 1500 people with CATI (computer assisted telephone interviewing) survey methodology in each month from the beginning of the pandemic. Through the combined analysis of the online and offline data, we evaluated the results of the large online survey and identified its most severe non-representative biases. To account for these biases, we developed a pipeline using iterative proportional fitting^43^ to weight the non-representative data in order to provide more representative contact matrices. This method supports the more realistic measurement of age contact matrices of a whole population while keeping the advantages (like cost-efficiency, scalability and detailed dynamics) of the online data collection. To describe our results, first we briefly summarize the structure and the content of the questionnaire and explain our data collection methods in details. Subsequently we introduce our methodology about the weighting of age contact matrices collected online. We found that the weighted online matrices resemble the best the representative observations if we consider dimensions partly derived from representative data collections conducted in the same period, and partly from the national census. We demonstrate the efficiency of the weighting procedure by presenting our methodology on contact matrices observed during the first wave of the COVID-19 pandemic in Hungary.

## Results

### Data collection

#### The MASZK questionnaire

The primary purpose of our questionnaire was to dynamically estimate the age contact matrices of people in different environments (like home, work, school, or elsewhere). For this very reason, we asked the respondent about the number of people from different age groups, with whom they had contacts with. First, we recorded *reference contact patterns* by asking respondents about their contacts during a typical weekday and weekend before the COVID-19 outbreak in Hungary (13th March 2020). Second, we recorded *actual contact patterns* of participants by asking them about their contact activities on the day before their actual response. We classified close contacts as *physical contacts* (direct physical contacts without using personal protective equipment), and *proxy contacts* (two persons stayed closer than 2 meters to each other at least for 15 minutes)^44^. Individual contact patterns were recorded as the approximate number of contacts between the ego and their peers from different age groups of 0–4, 5–14, 15–29, 30–44, 45–59, 60–69, 70–79, and 80+. For the sake of potential adoption of our method and reproducibility of our results we share the core part of our questionnaire including the essential questions for our analysis in the Supplementary Information (SI) and an online repository^45^.

**Figure 1:**
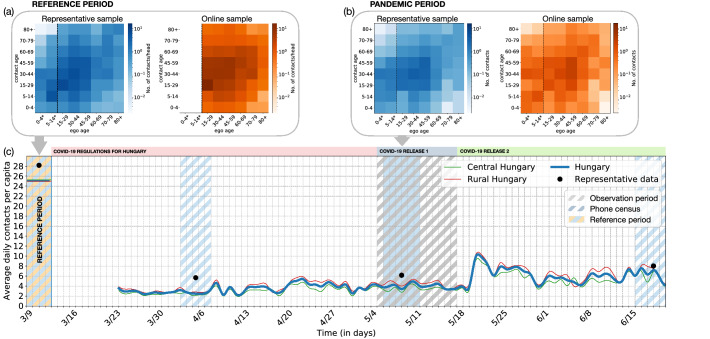
Contact dynamics, representative and non-representative age contact matrices. Age contact matrices measured during the (**a**) reference and (**b**) pandemic period via CATI survey methodology on a representative sample (blue) and via non-representative online data collection (orange) (for methodology see section on Construction of age contact matrices). Data for children under 18 (indicated with asterisk and vertical dashed lines) could not be collected directly due to privacy regulations, thus our data cannot provide a representative sample for the first two age groups. Their contact patterns are calculated based on the adult respondents’ responses on their children’s behavior. For the reference period at the online survey children’s contact patterns were not asked from their parents, therefore we do not have data on children’s contact patterns before the pandemic in the online sample. (**c**) Timeline of early pandemic regulations in Hungary and the average number of per capita daily proxy social contacts in rural areas (solid green line), the central area (red solid line) of Hungary, and in the whole country (blue solid line). While online data collection was continuously ongoing after the 23rd March 2020, representative data via telephone surveys were collected during the periods assigned by diagonal shading. Blue shades indicate telephone-based data collection (representative survey), while grey shades cover the online observation period of the actual study. Both methods retrospectively recorded the contact patterns about the reference period (before 13th March 2020), except for age groups under 15 in the online questionnaire.

#### Online data collection

MASZK was originally developed as an online survey^39^, and later it was also published as a mobile phone application^46^. Participation was and still is voluntary and the data collection was completely anonymous (for further details see the Methods section). The data collection started on the 23rd March 2020 and is still ongoing (as of March of 2022). While keeping the core questionnaire (shared in the SI) intact, the additional content has been adjusted to the actually pressing issues of the pandemic, like work and home office conditions, job security, self-protection practices, or intention for vaccination. Respondents were asked to fill out the questionnaire as many days, as they can, providing ongoing relevant information about their contacts. Up to date, the questionnaire has been completed in 515, 254 times by 234, 503 respondents, which accounts for $\sim 2.3\%$ of the population of Hungary. The collected data sensitively reflects public awareness and reactions to national regulations as it can be followed in Fig. 1c. During the reference period, until the 13th March 2020 when the first regulations were announced, the average daily number of proxy social contacts of individuals was measured $\sim 25$. This number dropped radically by $88\%$ to a value $\sim 3$ after a national lock-down was introduced. Subsequently, the lock-down was lifted first in rural Hungary (4th May 2020) and later in the more densely populated central region (18th May 2020). This was followed by a modest increase in the number of social contacts to $\sim 8$, which though never reached its reference value until the end of the observed period (20th June 2020). In this work, to demonstrate our methodology, we analyse a period of consecutive three weeks (29th April to 19th May 2020) during the first relaxation of the restrictive measures, as both types of data collection campaigns were conducted in these days. Using online surveys we recorded 30, 770 responses from 12, 208 people during this three-week period (see Methods, and SI, Table S1).

#### Nationally representative telephone survey

Additionally to the ongoing online data collection, CATI surveys were conducted to ask the same questionnaire on a nationally representative sample in each month. The sample size was 1, 500, which is $50\%$ larger than the conventional sample size for nationally representative samples in Hungary. Data collection campaigns were conducted in the beginning of the lock-down period (2–7 April 2020), during the first relaxation period (6–12 May 2020), and in each month after May 2020. In the current work, we analyze the data of the second period, where two-third of the data was collected about weekdays, while one third about weekends (for further details see Methods). Our goal with this data collection method was to obtain more realistic and representative data about the contact patterns of the Hungarian population; and to compare similar data coming from different sources to develop tools for reducing biases inherent in the non-representative sample of voluntary online survey. To demonstrate the differences between the representative telephone survey and non-representative online data, we show the average contact numbers computed from these two types of data (shown respectively as black points and blue solid line in Fig. 1c). It is evident that the online contact numbers systematically underestimate the values computed from the representative survey data.

### Construction of age contact matrices

In order to construct the age contact matrix of social contacts for the whole population, we collected information about the number of proxy and physical contacts of each respondent *x* during the reference and actual periods in different settings. For a given social connection type, period, and setting, using the age of the respondents we assigned them into one of eight age groups *A* (as defined in section The MASZK questionnaire), while doing the same for their contacts too. Thus we received an individual contact matrix $\mathrm {M}^x$ coding for each user *x* the number of contacts they had with others from age groups $i\in A$. Assuming an individual representative weight $w^x$ for each respondent, we computed a weighted average contact matrix $\left( {\mathbf {M}}\right) _{ij}$, which was column-wise normalised, thus giving us the weighted average number of contacts between a person from age group *j* with someone from age group *i*. Note that this matrix is not symmetric, and in case of a fully representative sample, weights would be $w^x=1$, simplifying the computation to a simple averaging process (see Methods). By comparing the age contact matrices computed from the representative telephone survey to matrices from the online data (see respectively blue and orange matrices in Fig. 1a and also in b), their evident differences indicate the consequences of the non-representativeness of the online sample, which we can account for by following the methodology explained below.

#### Social-demographic biases

Despite the many advantages of open online surveys, due to voluntary participation they often record a highly non-representative sample of the observed population, which may cause misleading conclusions about the nature of the epidemic process. To identify the most relevant social-demographic dimensions along which the online survey data is biased, we compare the non-representative online data to the corresponding national census.

**Figure 2:**
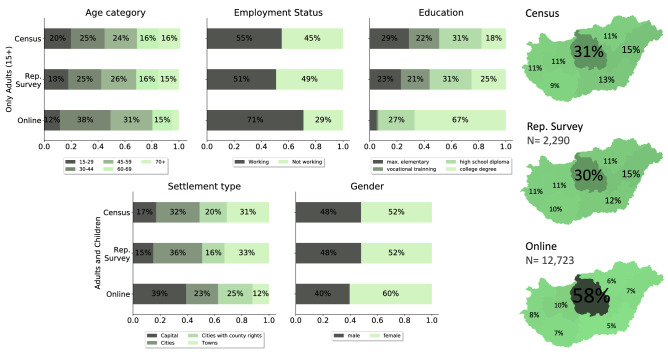
Descriptive statistics of key demographic variables. Variable statistics of the raw online data in the representative and in the online survey, compared to the population data of the Hungarian Statistical Office^47^ (Census) by the main socio-demographic variables age, employment status, education, region, settlement type, and gender. Note, that statistics showed for age category, employment status and education are based on the adult population of Hungary (15 years old or older), while settlement type, gender and regions covers the entire population.

Statistics shown in Fig. 2 evidently demonstrate that while the distributions of the nationally representative phone survey shows very similar values to the population census data provided by the Hungarian Statistical Office^47^, the online survey presents strong biases along the main socio-demographic dimensions. Compared to the census data, those who filled out the online survey are more likely to be middle aged, employed, higher educated, live in the capital and more likely to be women. On the other hand, people who are lower educated, older than 70 years, or live in small settlements like towns are under-represented. These striking differences suggest that the analysis of the raw online survey would lead to biased contact patterns, which are hardly generalizable for the whole Hungarian population.

#### The weighting procedure

The general method for handling the biases of a non-representative survey is to use correction weights based on the main socio-demographic variables of the population. However, in this case, where we aim to reproduce the contact patterns of people, those characteristics are relevant, that are significantly correlated with the number of contacts respondents had during the pandemic/after the lock-down was introduced. Since we want to select variables that have a direct (and not latent or casual) relationship with the number of contacts, we calculate Pearson’s correlations to select those variables, which significantly affect the number of contacts. Table 1 shows the Pearson’s correlation coefficients between the total number of proxy contacts and the earlier mentioned basic socio-demographic characteristics. All the correlations appear to be rather weak, but employment status and age. Thus, going beyond conventional socio-demographic dimensions, we selected other variables that might correlate with the contact patterns of the respondents. In the representative sample, three significant and strongly correlated factors were found: if the respondent has been in another city or district on the previous day; the number of people the respondent lives together with; and the typology of working on the previous day of the data collection, which contains the following categories: work from home, work from a workplace, did not work. Although the latter is quite similar to the one of employment status, the correlation coefficients suggest that there are major differences in the contact patterns between those, who work from home and those, who have to go to their workplace. After the detection of this combined set of significant variables, we apply a weighting methodology on the online survey to ensure that the online sample more accurately reflects the contact patterns of the whole population. The goal of this procedure is to provide an individual weight $w^x$ for each respondent *x*, which indicates how much they are needed to be taken into account in the re-constructed online data to make it more representative to the population from the aspect of contact patterns. Those respondents, who belong to a social group, which is underrepresented in the online sample get higher weights, while those from over-represented groups get lower ones. Depending on the difference between the composition of the online sample, and the population or representative data in terms of weighting variables, individual weights can take on a wide range of values, which can be undesirable as extreme weights can result unstable estimations^48^. Figure 2 shows that differences between the online and the census data are quite large (for a comparison of the online and representative data see SI, Table S2.). Therefore, to account for these large differences, our weighting methodology needs to meet two goals: bringing the contact patterns of the online survey data closer to the representative survey; while keeping the size of the weights in a reasonable range. To meet the second goal, we applied *iterative proportional fitting* (IPF), which, compared to standard cell weighting, is less likely to result extremely small or large weights. IPF is a weighting methodology, which adjusts the inner cells of an *n*-dimensional contingency table in a way that it returns the previously provided expected row and column margins^43^. In case of socio-demographic weighting dimensions, the expected margins (the distributions of the weighting variables) are taken from census data, while in the case of the three other variables, the expected margins are taken from the representative survey as no population data is available about their distribution (see Table S2, in SI for raw margins of the online data compared to census and the representative offline survey).

**Table 1:** Pearson Correlations Coefficients between the total number of proxy contacts of respondents in the representative survey and variables capturing their socio-demographic and life-style characteristics.

Pearson correlation coefficients with the total number of proxy contacts	
Works (1-yes, 0-no)	− 0.173***
Region: Central Hungary	0.019
Region: Southern Transdanubia	0.021
Region: Northern Great Plain	0.018
Region: Northern Hungary	0.001
Region: Central Transdanubia	− 0.001
Region: Western Transdanubia	0.031
Region: Central Hungary	− 0.060*
Settlement type: capital	− 0.055*
Settlemen type: city with country rights	0.016
Settlement type: city	− 0.009
Settlement type: town	0.042
Gender	− 0.049
Age	− 0.113***
Highest level of education	0.063*
Household size	0.127***
work typology: working in home office	− 0.020
work typology: must go to workplace	0.244***
work typology: does not work	− 0.199***
Student in higher education (1-yes, 0-no)	− 0.016
Works in healthcare (1-yes, 0-no)	− 0.046
Has been in abroad in the last month (1-yes, 0-no)	− 0.048
Has been in another city or district on the previous day (1-yes, 0-no)	0.123***

Stars (*) indicate the level of significance.

*p*< 0.05, **p< 0.01, *** *p*< 0.001.

To obtain well fitting weights, which satisfy both of our goals, we tested three strategies. In the first strategy, we only included the basic socio-demographic variables from census in the weighting procedure. In the second strategy, we only included those three survey variables, which were detected by the correlations of the variable selection process. In the third one, we combined the socio-demographic and the survey based variables (see SI, Table S2 for detailed information about variables used in the weighting procedure). Note, that as the contact matrices are built up by age-group-wise normalized vectors for each age group, the relative proportions of age groups can be neglected (which means that we do not include them as expected margins) in the IPF, which considerably decreases the variation of the obtained individual weights. In all three cases, we optimized the maximum values of weights (for details, see SI, Section *Weight optimization*) and calculated their efficiency. We used three measures for the quantification of efficiency. Our most important metric to evaluate the weighting procedure is the Relative Accuracy Gain (*RAG*). It quantifies how much we gain in terms of accuracy when we calculate the difference between the representative contact matrix and the weighted contaxt matrix, as compared to the difference between the representative and the unweighted case. It is defined as the function of the sum of absolute differences in the total number of contacts between the representative and the weighted online and the representative and not weighted online matrices (for exact formula see Methods). This metric is calculated for adults only as well, due to limited information available about respondents under 18. Second, we computed the sum of the contact errors of the weighted online matrix compared to the matrix of the representative survey (*SCER*). Third, we calculated the sum of contact error differences between the weighted and the non-weighted online survey’s matrices (*SCED*) (the results of the efficiency tests are presented in SI, Fig. S2., and Table S3, for formulas see Methods/Evaluation Metrics). The goal of the optimization procedure is to maximize the *RAG*, while keeping *SCER* and *SCED* as low as possible. In all three measures, the least efficient strategy was the one with only the socio-demographic variables extracted from census - which underlines the importance of additional factors. Even the strategy, which only included the survey-based three variables showed better results. Nevertheless, the most efficient strategy was the one, where we combined the socio-demographic and the survey based variables. In the following, we will analyze the results of this weighting strategy.

Compared to standard cell weighting, IPF is less likely to result extremely small or large weights. The optimization yields that $w_{max}=2$ using both the survey and the census data performed the best, with individual weights distributed over a relatively small range, between $0.01<w^x<2$. (See distribution SI. Fig. S3). The low value of the maximum weight with this level of efficiency is especially remarkable - taking the large differences of the online and representative/census data into account. The closer an individual weight is to one, the more the corresponding individual is representative of their group - by the listed variables.

**Figure 3:**
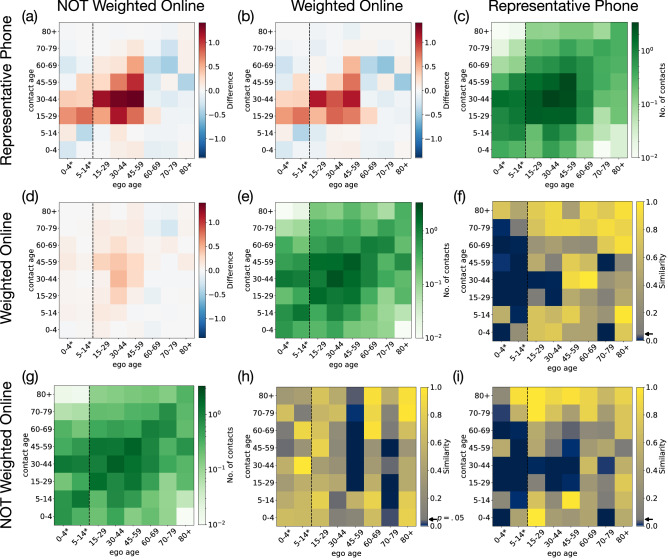
Results of iterative proportional fitting. Normalized actual proxy contact matrices (green diagonal), their pairwise difference matrices (above diagonal) and pairwise two-tail T-test results (below diagonal) are depicted for the online non-weighted, online weighted, and representative matrices. In the difference matrices red or blue cells indicate that the source matrix (column label) appeared with higher or lower number (respectively) of average contacts compared to the target (row label) in a given cell. For results of pairwise two-tail T-tests blue to yellow cells (corresponding to $p>0.05$, assigned by an arrow beside the colorbar) indicate that the given cell is not significantly different in the source (column label) and target (row label) matrices. Data for children under 18 (indicated with asterisk and vertical dashed lines) could not be collected directly due to privacy regulations, thus our data cannot provide a representative sample for the first two age groups (see Limitations).

### Reconstructed matrix analysis

In this subsection, we analyze the efficiency of the selected weighting procedure, taking the representative contact matrix as a reference point and comparing the weighted and non-weighted contact matrices with it - and also with each other. Note that due to data protection regulations, the representative data collection is only representative for the adult population of Hungary, thus it only worth to focus on the 15+ years age groups of egos in the analysis of the contact matrices. We separated the two younger age groups with a dashed line on Fig. 3 to visualize this distinction. The data collected for children is originated from the respondents, who provided approximation of the contacts of their children. For children’s data we used a more simple weighting procedure, with methodology explained in the SI (see Section Weight optimization). The reconstructed online proxy age contact matrix (panel Fig. 3e) appeared with an expected structure very similar to the representative result (panel Fig. 3c). It exposes a strong diagonal component induced by age homophily (for annotated matrices see SI, Fig. S3), meanwhile it suggests larger contact numbers between people of age 15-59, including the employed population of the country. These matrices were recorded during the period in May 2020, when schools were closed in Hungary. This is reflected in the higher contact numbers between the youngest age groups and their parents’ generation from the age group of 30-44. However, if we compare the representative matrices to their corresponding reference period measures (see Fig. 1a and b), we evidently see the radical decrease in the number of contacts (darker shades for reference period and lighter for the later one). Through this comparison it is also evident, that the closure of schools significantly reduces the number of homophilic contacts between children of age 5-14 as compared to the reference period.

To quantify the precision of our reconstruction method we compare the raw (not weighted) and reconstructed online proxy contact matrices to the corresponding representative matrix to see, which age cells changed the most, and which of them became closer to their representative value due to the the reconstruction. In the diagonal of Fig. 3 we depict the three actual proxy contact matrices built from the representative survey (Fig. 3c), from the reconstructed (weighted) online survey (Fig. 3e) and the raw (not weighted) online survey (Fig. 3g). First, in the upper diagonal, we compare these matrices by calculating their pairwise differences (see Fig. 3a, b and d). The difference between the representative survey and the raw online data (Fig. 3a) shows that middle-aged respondents of the online data collection had lower number of average contacts with young and middle aged adults than the respondents of the representative survey. Meanwhile, the non-representative online data collection overestimates the number of contacts of elderly people with others of similar age. However, while the absolute difference in the total number of contacts of the 15+ years age groups (for which the representative survey is comparable) between the representative and the not weighted online survey was 13.8, after reconstruction this difference between the representative and weighted online matrices reduced to 10.7. This corresponds to a $18.37\%$ increase in Relative Accuracy Gain considering only adults ($RAG=14.23$ in case age groups below age 15 are counted). The difference matrix of the non-weighted and weighted matrices depicts the effect of the reconstruction process on the online matrix (see Fig. 3d). Although the magnitudes of differences are not large, certain heterogeneities are visible, like the decrease of contact numbers between middle age people and the increase of contacts between 70-79 years old egos and similar others after the reconstruction.

To further quantify the goodness of the weighting in detail, we tested if a cell of a contact matrix is significantly different from the same cell of another contact matrix. Each cell of a contact matrix ${\mathbf {M}}_{ij}$ appears as the average of the distribution of the number of contacts between the age-group *j* of a respondent and the age group *i* of their peers. Thus we can perform a pairwise two-tailed independent sample T-test for each cell to see whether the population means of two groups corresponding to respective cells measured in different contact matrices are significantly different from each other^49^. These tests show if the differences between the average contact numbers of various data sources (presented in the upper diagonal of the figure) are statistically significant.

In the visualisations of the lower diagonal panels of Fig. 3, yellow cells correspond to $p>0.05$ values ($p=0.05$ is indicated by arrows near colorbars) suggesting that average contact numbers between the corresponding age groups are *not* significantly different in the two data sources. To check the robustness of our matrix reconstruction method, we performed the same significance test between the raw (not weighted) online matrix and the representative matrices (Fig. 3i). Comparing its results to the results of the weighted and representative matrices (Fig. 3f) among the 15+ age groups (for which the representative survey is valid), the number of cells, which are not significantly different increased by $11.6\%$ in the latter (from from 38 to 43), while the range of similarity has also elevated (indicated by more yellow cells). For example, this is the case for the cell between 45-59 year old egos and 15-29 year old peers where the difference between the non-weighted and representative matrices is significant (see Fig. 3i), but due to the weighting procedure their difference reduced and became non-significant (see Fig. 3f). Meanwhile, from the T-test results between the raw (not weighted) and weighted online matrices (see Fig. 3h) it is evident that the weighting helped to capture the contact patterns better in the reconstructed matrix, especially in case of the active population (45–59), and the 70–79 years old with younger others. Precise estimation of the contact patterns of these age groups are especially important for predicting the potential number of infected cases, which may end up with severe medical conditions in case of the COVID-19 pandemic^23^. These results show that the reconstruction caused significant changes in the values of 5 cells out of the 48 and that these changes brought the value of the given cell closer to the representative one in most cases (for exact significance values see SI, Fig. S3).

#### Limitations and future directions

One potential limitation of the data collection was the validity of the responses due to possible recall bias at the contact related questions. As these questions were asked retrospectively (the period before COVID-19 and the day before the data collection), it is possible that respondents could not recall their memory accurately, and thus, did not give accurate answers for the questions. Recall bias can be present for many different reasons, some of which are intentional (e.g., social desirability bias), some of which are not (e.g., the natural way how our brain works and edit our memories). Observed in many fields^50–52^, the results of retrospective surveys are frequently biased compared to the actual incidences. In our cases, we can assume that answers about the proxy contacts of respondents before the pandemic were more biased in this sense, while their recall was more precise when answering questions about the day before. Thus, although we cannot exclude the presence of recall bias, as the past date we ask the respondents about is relatively close, we can assume that their effects were limited.

It is very important to emphasize that the comparison of the actual proxy contacts in the representative and weighted/not weighted online matrices does not follow the same logic for children in the first two age groups. Due to data protection regulations, the CATI survey is only representative for the adult population of Hungary and not for children, while the online survey could not involve underage children either. As we have mentioned earlier, data of children are based on the responses of adult parents estimating the contact patterns of their own children. This estimation is surely biased as, especially for older children, parents may not be fully aware about all daily social contacts of their children. Consequently, we cannot use the representative sample as a ’gold standard’ for these age groups, because the population of children recorded in that data is not representative for the children population of the whole country. Correction of this bias would require a separate data collection campaign involving a representative set of children directly, which in turn would raise challenges to meet privacy regulations of under-aged participants and fall beyond the scope of the actual study. Nevertheless, this explains the larger differences between the online and representative matrices in the first two columns in Fig. 3 off-diagonal panels. To make this bias evident, we separated the non-representative age groups with a vertical dashed line within the matrices, while indicated by asterisks at the labels in each relevant plot.

Treating survey data as the ’gold standard’ in the evaluation of the weighting procedure is not an evident decision. The survey data itself could carry potential bias resulting for example from sampling error or social desirability bias. Although these biases can be present in the representative survey data, out of the available data collections, this can bring us the closest to the real behaviour of a whole adult population of a country. Meanwhile, we regard non-response bias to be low in our case, as the response rate was rather high ($49\%$), which can be explained by the high interest in the current topic of the questionnaire. It is important to mention, that in all waves of the data collection, the data collected by the phone survey were always representative for the Hungarian adult population along the dimensions we discussed in the paper, independently of the percentage of non-response.

Another potential limitation may be rooted in the sampling of the observed population. This issue is present at the online data collection, where the number of responses may vary in time. If the size of the online sample is too small or if the composition of the online sample changes, individual weights could diverge and the reconstructed matrices would suffer from large errors. In the present study, this is not an issue, as in the examined period the number of daily responses were stable and relatively high. However in the case of a longitudinal data collection, these parameters can change due to the varying level of public awareness, political influence, or media campaigns.

Finally, not only the number, but also the factors, which affect the number and pattern of contacts may change in time, thus the efficiency of the actual weighting procedure may decrease. To account for this effect in the dynamical reconstruction of contact matrices, one would need to make a representative data collection periodically, and recompute the relevant dimensions (and weights) for each period. Although we have collected representative samples in each month since April 2020, the demonstration of dynamical re-weighting is the subject of a future investigation (in preparation).

## Discussion

Emergency situations, like the actual COVID-19 pandemic, may induce radical changes in the behavioural patterns of people leading to the reduction and re-organisation of their social interactions^53^. Changes may be induced by external influence such as governmental interventions, or change in employment status, but they may strongly depend also on individual decisions induced by self-, and environment-awareness or risk avoiding behaviour. All these influence have convoluted effects on the size and structure of personal interactions leading to different paths of epidemic transmissions in a connected population^54^. Age contact matrices provide a useful way to summarise and follow such changes in the social fabric at different settings and time. Importantly, they can be further used for more realistic modelling of epidemic spreading. Nevertheless, their collection was rather spurious, expensive, and other than some recent studies^21,23,37,38^, they were collected during ’normal’ times, thus they commonly missed to capture changes in contact patterns during emergency periods.

The aim of this work was to provide a methodological framework for a feasible alternative approach, which combines the advantages of large scale online data collections with the accuracy provided by a cross-sectional and smaller scale representative telephone survey. We report here, one of the largest data collected to date to estimate age contact matrices in a single country, reaching over $2.3\%$ of the population of Hungary. As the online data provided a non-representative sample of the population, we developed a methodology to reconstruct closer-to representative contact matrices from the online data by using the simultaneously collected representative samples. As compared to other methods developed, like the earlier mentioned personal device based mobility data^6^, or newly developed contact tracing apps^55–57^, our methodology suggests a dynamical, representative, and anonymous data collection, which provides accurate information on people’s contact-related behaviour on the personal level This data collection method is not only scalable, flexible in terms of content, and relatively cheap, but it also allows for dynamical estimation of contact matrices with high temporal and spatial resolution.

The reproducibility of our results and the possible adoption of our methods in different countries are primary concerns for us. For these reasons, along this study, we share the core questionnaire for further use^45^, together with the raw, reconstructed, and representative matrices and all supporting data calculated for Hungary. Up to date, our data collection method has been implemented already in Mexico^58^ and Cuba. We hope that it will prove useful to collect relevant data for applied epidemiological modelling in other countries too, and at large, will contribute to the global efforts to fight the actual COVID-19 and any future pandemic.

## Materials and methods

### Data collection

#### MASZK online data collection

The online data collection started on the 23rd of March 2020 through the website covid.sed.hu and later using a mobile phone app^46^. We obtained fully informed consent from every participants before enrolment in the study. The anonymity of participants was ensured by using encrypted browser cookies to store hashed identifiers locally, while transferring only anonymous encrypted data to a central secure server. Encrypted browser cookies were used for the detection of returning respondent filling out the questionnaire on multiple days. The participants did not have to give any information, which could be used for their re-identification. The data collection was fully complying with the actual European and Hungarian privacy data regulations and was approved by the Hungarian National Authority for Data Protection and Freedom of Information^59^, and also by the Health Science Council Scientific and Research Ethics Committee (resolution number IV/3073- 1 /2021/EKU). During our analysis all methods were performed in accordance with these relevant guidelines and regulations. The data collection is accompanied with an ongoing marketing campaign, including regular radio and newspaper interviews, ads on social media platforms, and posters on public transportation, to reach the broadest audience possible. Targeted campaigns were also published with help of national organisations to reach parents, university students, or elderly people.

In this study, we analyse data collected between the 29th of April and the 19th of May 2020 and recorded 30, 770 responses from 12, 208 respondents of the online questionnaire. The questionnaire was constructed by two parts in order to minimise the burden and potential churning (sample attrition) of participants:

##### Static questionnaire

It was asked only once upon first response (controlled by encrypted browser cookies) about information, which do not change frequently, like the year the respondent was born, gender, domicile, education level, etc. This static part also included questions about the proxy contact patterns of the respondent during the *reference period*, before the official declaration of the pandemic, 13th of March 2020. We recorded reference contact patterns separately for typical weekdays and weekends of the respondents together with their age and gender detailed household structure.

##### Dynamic questionnaire

It was asked to be completed ideally on a daily basis about the activities of the respondent on the previous day. More specifically, we asked the reasons they were outside, the places they visited, the protections they wore, travel mode they used, the changes in their working conditions, etc. We asked questions about their *proxy* and *physical* social contacts outside their home, at work, or elsewhere; and also about those people, with whom they had contacts at home, but who are not part of their household. For those, who mentioned children under 18 years in their household, more questions were asked about the contact patterns of their children at school or elsewhere. We share the full questionnaire including the essential questions for our analysis in the SI and an online repository^45^.

#### Nationally representative CATI survey

A smaller scale, but nationwide representative data collection was also conducted between the 6th and 12th of May 2020 using exactly the same questionnaire taken from the online survey. The data collection was implemented by CATI survey methodology. A multi-step, proportionally stratified, probabilistic sampling procedure was elaborated and implemented by the survey research company using a database that contained both landline and mobile phone numbers. The response rate was 49 percent, which is expressly higher than the average response rate of telephone surveys in Hungary (and also in other countries), and which can be explained by high interest in the quite current topic of the questionnaire. (According to the data collection company, the average response rate of this data collection methodology at a nationally representative survey is between 15-20 percent.) The sample is representative for the Hungarian population aged 18 or older by gender, age, education and domicile. Sampling errors were corrected using iterative proportional post-stratification weights. After data collection, only the anonymised and hashed data was shared with people involved in the project after signing non-disclosure agreements.

### Contact matrix construction

We categorised people into eight age groups, as defined in the main text, thus constructed $8\times 8$ matrices with column indices corresponding to the age group of our respondents and row indices correspond to the age group of their contacts. In order to compute the population level age contact matrix, we use a formal description. Let *X* be the set of respondents (ego), and let *Y* be the set of individuals who are contacts of some $x\in X$. For a specific *x*, let $N_x \subset Y$ be the set of individuals who are contacts of *x*. We assign by $a(x)\in A=\{1,\dots ,8\}$ the age group of an individual *x*. Next we define the matrix $M^{x,y}$ for each $x\in X$ and $y \in N_x$ as follows: $\left( M^{x,y}\right) _{i,j}=1$ if $a(x)=j$ and $a(y)=i$, and zero otherwise. For an ego *x* we can now compute its individual contact matrix as $\mathrm {M}^x=\sum _{y\in N_x} M^{x,y}$. Finally, we use an individual weight $w^x$ assigned to each ego, coming from the IPF weighting method described in the main text. This weight effectively describes how much an ego and its contacts should be considered in order to receive a contact matrix for a closer-to-representative population. The population level contact matrix is computed by

$$\begin{aligned} \mathbf{M }=\sum _{x \in X} w^x \mathrm {M}^x \big / \sum _{x \in X} w^x. \end{aligned}$$

### Evaluation metrics

${\mathbf {M}}_{rs}$ denotes the actual proxy matrix obtained from the nationally representative survey, ${\mathbf {M}}_{ow}$ is the weighted actual proxy matrix obtained after reconstruction from the online survey, and ${\mathbf {M}}_{onw}$ is the not weighted actual proxy matrix measured directly from the online survey.

We define **Relative Accuracy Gain (***RAG***)** in our setting to quantify how much we gain in terms of accuracy to approximate the representative contact matrix due to the weighting procedure of the online contact matrix, as compared to the unweighted case. It is defined as the function of the sum of absolute differences in the total number of contacts between the representative (rs) and the weighted online (ow) and the representative and not weighted (onw) online matrices. More formally

1 $$\begin{aligned} RAG = 1-\left( \frac{\sum |{\mathbf {M}}_{rs}-{\mathbf {M}}_{ow}|}{\sum |{\mathbf {M}}_{rs}-{\mathbf {M}}_{onw}|}\right) . \end{aligned}$$

We define the sum of Contact Errors compared to Representative matrix (*SCER*) as the sum of the contact errors of the weighted online matrix compared to the matrix of the representative survey (*SCER*). More formally,

2 $$\begin{aligned} SCER = \sum |{\mathbf {M}}_{rs}-{\mathbf {M}}_{ow}|. \end{aligned}$$

We define Sum of Contact Error Difference (*SCED*) as the difference of the sum of contact errors between the weighted and the non-weighted online survey’s matrices (*SCED*). More formally,

3 $$\begin{aligned} SCED = \left( \sum |{\mathbf {M}}_{rs}-{\mathbf {M}}_{onw}|\right) -\sum |{\mathbf {M}}_{rs}-{\mathbf {M}}_{ow}|. \end{aligned}$$

## Supplementary Information

Supplementary Information.
