# Supplementary material for: Reconstructing social mixing patterns via weighted contact matrices from online and representative surveys
Source: Sci Rep. 2022 Mar 18;12:4690. doi: 10.1038/s41598-022-07488-7 (PMC8931780; doi:10.1038/s41598-022-07488-7)
Supplement: Supplementary file 1 — Supplementary Information. [file 41598_2022_7488_MOESM1_ESM.pdf]

Supplementary Information

Reconstructing social mixing patterns via weighted contact matrices from online and representative surveys

Júlia Koltai, Orsolya Vásárhelyi, Gergely Röst and Márton Karsai

corresponding authors: Márton Karsai (karsaim@ceu.edu)

1 The MASZK Hungarian Data Provider Questionnaire

The goal of the Hungarian Data Provider Questionnaire (MASZK) was to dynamically estimate the age contact matrices of people in different settings (like home, work, school, or elsewhere). To collect such data we developed a questionnaire to ask about people's demographic characters, domicile, family structure, health conditions, travel patterns, education level, employment situations and many more. More importantly we asked them about the number of people from different age groups, with whom they had contacts. First, we recorded *reference contact patterns* by asking respondents about their contact patterns during a typical weekday and weekend before the COVID-19 outbreak in Hungary (13th March 2020). Second, we recorded *actual contact patterns* of participants by asking them to indicate all their contact activities happened on the day before their actual response. We defined contacts in two different ways relevant for possible infection transmission. Interactions between people without any protection were called *physical contacts*, while *proxy contacts* were identified as if two people stayed closer than 2 meters to each other at least for 15 minutes. Individual contact patterns were recorded as the number of contacts between the ego and their peers from different age groups of $0-4$, $5-14$, $15-29$, $30-44$, $45-59$, $60-69$, $70-79$, and $80+$. Due to privacy regulations, contact patterns of under-age respondents was not possible directly. Nevertheless, to collect data about children younger than 18 years old, we asked respondents living in the same household with an under-age children to estimate their number of contacts in different settings. For the sake of potential adoption of our method and reproducibility of results we share the questionnaire including the essential questions for our analysis in this repository[1].

	**N**		
All respondents	13,790		
Children* (0-14)	1,582		
Adults (15+)	12,208		
**Age groups - weighting**	**N**	**N after weighting**	*Missing*
0-14	1,582	1,527	*55*
15-29	1,617	1,286	
30-44	4,423	4,152	
45-59	3,880	3,596	
60-69	1,712	1,611	
70+	576	551	*1,012*
**ALL**		**12,723**	***1,067***

Table S1. Number of responses after filtering users with not available data points. The original dataset contains responses from adults and for children based on parents responses. We applied a weighting methodology called iterative proportional fitting on the online survey to make it more accurate of measuring the contact patterns of the whole population. Since this methodology requires each variable used in the weighting to have non zero entry, we had to drop respondents with no data about variables used in the weighting procedure. See Table 2 for more details about variables used in the weighting procedure.

1.1 Response dynamics

As explained in the main text, the online data collection started on the 23rd of March 2020 through the website covid.sed.hu and later using a mobile phone app[2]. Respondents were asked to fill out the questionnaire as many days as they can, to provide ongoing relevant information about their contacts. The questionnaire was very popular after its release as reflected by the very large number of responses in the beginning of data collection (see Fig. 1a blue line). Nevertheless, after this initial period, the number of filled questionnaires settled around 1000 daily responses. At the same time, the daily number of new respondents kept decreasing, and settled around 20 person per a day (see Fig. 1a orange line). This high rate of return of respondents is well demonstrated by the distribution of number of responses per respondents (see Fig. 1b). It

indicates that although most of the respondents submitted only one response, the broad tail of the distribution shows that several users filled the questionnaire multiple times, with a maximum of 96 number of responses.

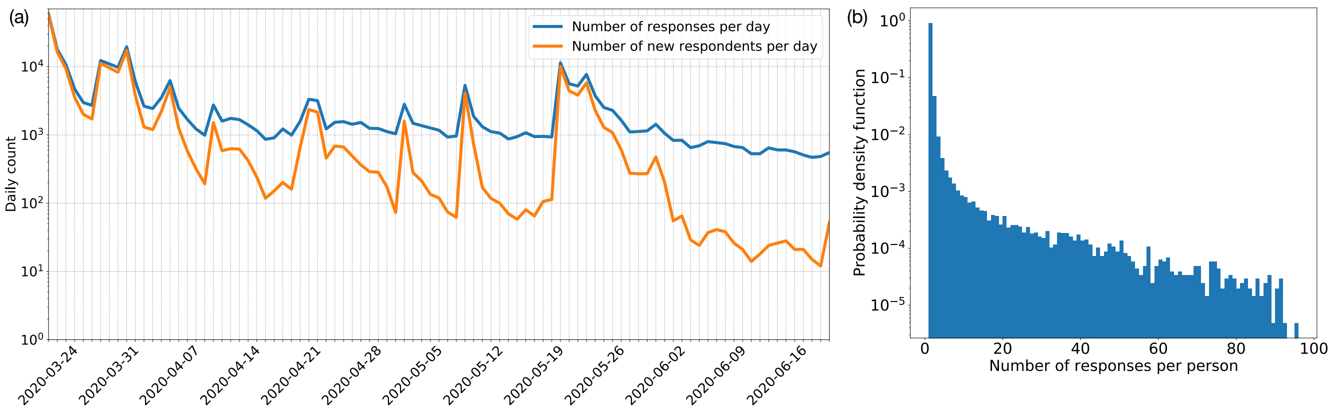

Figure S1. Response rates of the online MASZK questionnaire. (a) Rate of daily responses (orange line) and rate of daily new respondents (blue line). (b) Distribution of the number of responses per user. All statistics have been computed for the period between 23rd March 2020 and 22nd June 2020.

2 Weight optimization

Using the detected census and survey based variables effecting significantly the contact patterns, in the MS we described a weighting methodology for the online survey. Our goal was to provide a method which assign a w^x weight to each individual x, which are not distributed very broadly, as extreme weights increases the standard errors of the estimates and decrease the accuracy of the estimation. Therefore, our weighting methodology needs to keep the weights in a reasonable range.

We ran *iterative proportional fitting* (IPF) with maximum weight as a parameter within the range of 2 and 40 (*MX* theoretical) using three different variable combinations: Variables and their margins come 1) from the national census, 2) from the representative survey, 3) national census and the survey together (survey+census). Table S2 shows the variable groups' margins by data source. Weights were calculated separately for adults and children. Results in Table S3 are grouped by variable groups (census,survey, survey+census) and the maximum weight parameter (*MX* theoretical). *MW* shows the realized maximum weight, which in the case of "census" stops at 1.93, that is why results are not changing regardless of theoretical maximum weight. Relative Accuracy Gain (*RAG*) quantifies how much we gain in terms of accuracy to approximate the representative contact matrix due to the weighting procedure of the online contact matrix, as compared to the unweighted case. It is defined as the function of the sum of absolute differences in the total number of contacts between the representative and the weighted online, and the representative and not weighted online matrices. *RAG* (only adults) is the same metric taking only into account adults. *SCER* is the sum of the contact errors of the weighted online matrix compared to the representative survey's matrix. *SCER* (only adults) is the same metric calculated for adults only. *SCED* is the sum of contact errors difference between the weighted and the non-weighted online survey's matrices. (For more details see MS Methods Section, Evaluation Metrics). The goal of the optimization procedure is to maximize *RAG*, while keeping *SCER* and *SCED* low.

For children we could use only 3 socio-demographic variables: age group settlement type and gender to generate weights applying also IPF. The range of weights of children is between $0.04 < w^x < 3.3$, which is still in the acceptable range.

Fig. S2) indicate that $w_{max} = 2$ using both the survey and the census data performed the best with a $RAG = 14.23$, and $RAG_{adults} = 18.37$, which resulted in individual weights distributed over a relatively small range, between $0.01 < w^x < 2$ as demonstrated in Fig. S3).

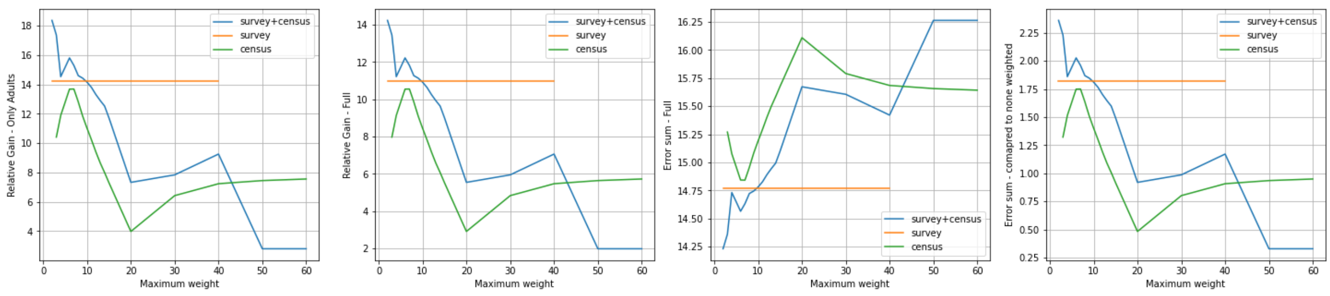

Figure S2. Accuracy metrics visualized to choose optimal weight and variable group.

.

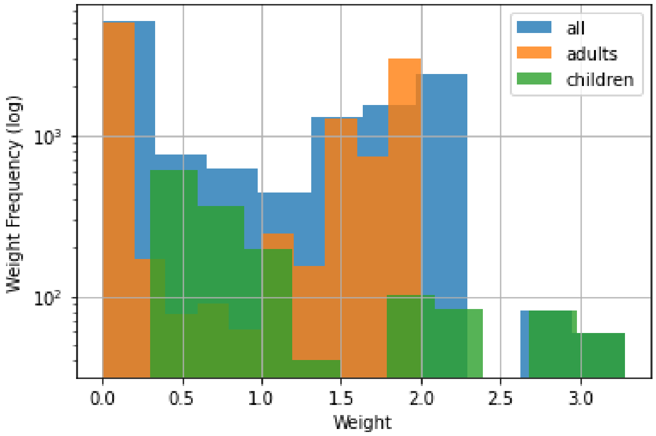

Figure S3. Resulting logarithmic weight distribution of iterative proportional fitting (IPF) for adults, children and the entire population. The maximum weight for adults is 2.00, and for children is 3.3. The obtained weights stayed within the range of 0.04 and 3.3, with an average of 0.92.

.

3 Annotated matrices

To extend our results reported in the main text, here we summarise the measured and reconstructed contact matrices and their comparison in a matrix plot panel, annotated with numerical values. More precisely, in Fig. S4 in the diagonal we show the representative, online-weighted, and online-unweighted matrices. Above the diagonal we depict the pairwise differences between these matrices, while below the diagonal we show the pairwise two-tail T-test results.

The raw, reconstructed, and representative matrices are shared as data tables in an online repositories[1].

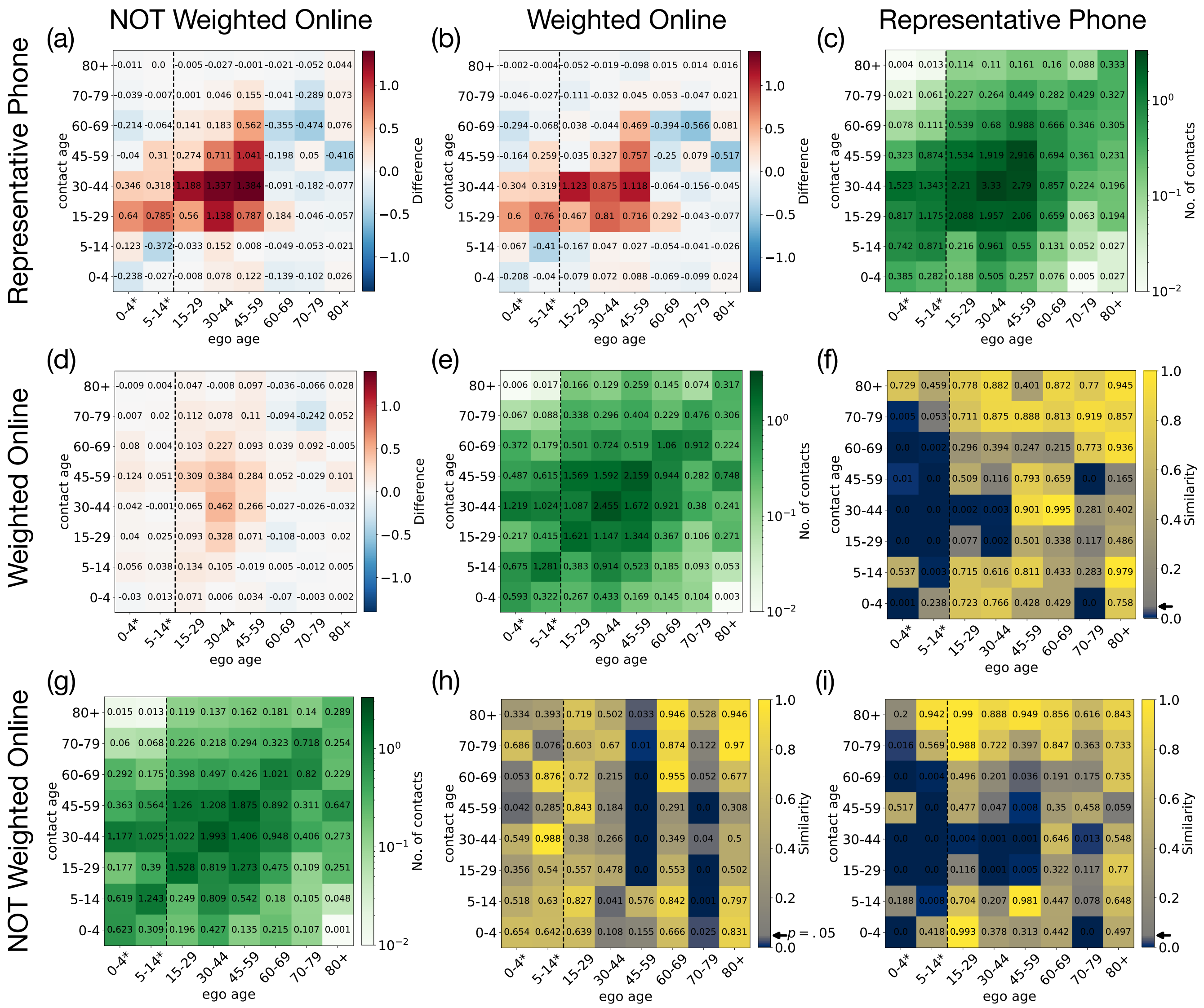

Figure S4. Annotated matrices. Normalized actual proxy contact matrices (green diagonal), their pairwise difference matrices (above diagonal) and pairwise two-tail T-test results (below diagonal) are depicted for the online non-weighted, online weighted, and representative matrices. In the difference matrices red or blue cells indicate that the source matrix (column label) appeared with higher or lower number of average contact than the target (row label) at the given cell. For results of pairwise two-tail T-tests yellow to blue cells (corresponding to $p > 0.05$, assigned by an arrow beside the colorbar) indicate that the given cell is not significantly different in the source (column label) and target (row label) matrices. Data for children under 18 (indicated with asterisk and vertical dashed lines) could not be collected directly due to privacy regulations, thus our data cannot provide a representative sample for the first two age groups.

ADULTS	Variable Name				
**Survey**	**Work Typology)**	**Home Office**	**Did not work**	**Went to the office**	
	Margin	12.8%	64.8%	22.4%	
	Data	3,778<br>(33.50%)	5,849<br>(51.86%)	1,651<br>(14.64%)	
	**Been in another city**	**No**	**Yes**		
	Margin	83.2%	16.8%		
	Data	8,919<br>(78.18%)	2,489<br>(21.82%)		
	**Household size**	**Lives alone**	**2 people**	**3 people**	**4+ people**
	Margin	20.5%	33.1%	19.9%	26.5%
	Data	2,147<br>(19.28%)	3,836<br>(34.44%)	2,203<br>(19.78%)	2,952<br>(26.50%)
**Census**	**Settlement Type**	**Budapest**	**City with county rights**	**City**	**Town**
	Margin	17%	20%	32%	31%
	Data	4,255<br>(38.86%)	2,778<br>(25.37%)	2,454<br>(22.41%)	1,462<br>(13.35%)
	**Region**	**Not Central Hungary**	**Central Hungary**		
	Margin	69%	31%		
	Data	4,140<br>(41.19%)	5,910<br>(58.81%)		
	**Gender**	**Male**	**Female**		
	Margin	48%	52%		
	Data	4,353<br>(39%)	6,905<br>(61%)		
	**Education**	**Max. elementary**	**Vocational School**	**High-School diploma**	**College degree**
	Margin	29%	22%	31%	18%
	Data	143<br>(1.30%)	446<br>(4.05%)	3,081<br>(27.98%)	7,342<br>(66.67%)
	**Work status**	**Working**	**Not working**		
	Margin	55%	45%		
	Data	7,947<br>(72.13%)	3,071<br>(27.87%)		
**CHILDREN**	**Age**	**0-14**	**15-18**		
	Margin	51.7%	48.3%		
	Data	1,563<br>(81.32%)	359<br>(18.68%)		
	**Gender**	**Male**	**Female**		
	Margin	51.4%	48.6%		
	Data	911<br>(50.28%)	901<br>(49.72%)		
	**Settlement Type**	**Budapest**	**City with county rights**	**City**	**Town**
	Margin	18%	18%	34%	36%
	Data	700<br>(36.38%)	446<br>(23.18%)	484<br>(25.26%)	292<br>(15.18%)

Table S2. Population census, and distributions from the representative survey for variables used for applying the weighting methodology called iterative proportional fitting on the online survey for adults and children to make it more accurate of measuring the contact patterns of the whole population.

Weighting procedure	MX (theoretical)	MW Realized	RAG (only adults)	RAG	SCER (Only Adults)	SCER	SCED
survey	2.00	1.93	14.24	10.98	11.20	14.77	1.82
survey+census	2.00	2.00	18.37	14.23	10.66	14.23	2.36
survey	3.00	1.93	14.24	10.98	11.20	14.77	1.82
survey+census	3.00	3.00	17.38	13.45	10.79	14.36	2.23
census	3.00	3.00	10.42	7.97	11.70	15.27	1.32
survey	4.00	1.93	14.24	10.98	11.20	14.77	1.82
survey+census	4.00	4.00	14.54	11.21	11.16	14.73	1.86
census	4.00	4.00	11.89	9.13	11.51	15.08	1.52
survey	5.00	1.93	14.24	10.98	11.20	14.77	1.82
survey	6.00	1.93	14.24	10.98	11.20	14.77	1.82
survey+census	6.00	6.00	15.81	12.22	10.99	14.57	2.03
census	6.00	6.00	13.69	10.54	11.27	14.84	1.75
survey	7.00	1.93	14.24	10.98	11.20	14.77	1.82
survey+census	7.00	7.00	15.30	11.81	11.06	14.63	1.96
census	7.00	7.00	13.69	10.55	11.27	14.84	1.75
survey	8.00	1.93	14.24	10.98	11.20	14.77	1.82
census	8.00	8.00	12.82	9.86	11.38	14.96	1.64
survey+census	8.00	8.00	14.60	11.26	11.15	14.72	1.87
survey	9.00	1.93	14.24	10.98	11.20	14.77	1.82
survey+census	9.00	9.00	14.44	11.13	11.17	14.75	1.85
census	9.00	9.00	11.85	9.09	11.51	15.08	1.51
census	10.00	10.00	11.02	8.44	11.62	15.19	1.40
survey+census	10.00	10.00	14.16	10.91	11.21	14.78	1.81
survey	10.00	1.93	14.24	10.98	11.20	14.77	1.82
survey	11.00	1.93	14.24	10.98	11.20	14.77	1.82
survey+census	11.00	11.00	13.81	10.64	11.26	14.83	1.77
census	11.00	11.00	10.24	7.83	11.72	15.29	1.30
census	12.00	12.00	9.42	7.18	11.83	15.40	1.19
survey	12.00	1.93	14.24	10.98	11.20	14.77	1.82
survey+census	12.00	12.00	13.33	10.26	11.32	14.89	1.70
survey	13.00	1.93	14.24	10.98	11.20	14.77	1.82
survey+census	13.00	13.00	12.91	9.93	11.37	14.94	1.65
census	13.00	13.00	8.66	6.59	11.93	15.50	1.09
survey+census	14.00	14.00	12.54	9.64	11.42	14.99	1.60
census	14.00	14.00	8.00	6.07	12.01	15.59	1.01
survey	14.00	1.93	14.24	10.98	11.20	14.77	1.82
census	15.00	15.00	7.31	5.52	12.10	15.68	0.92
survey	15.00	1.93	14.24	10.98	11.20	14.77	1.82
survey+census	15.00	15.00	11.73	9.00	11.53	15.10	1.49
survey+census	20.00	20.00	7.33	5.54	12.10	15.67	0.92
census	20.00	20.00	3.98	2.91	12.54	16.11	0.48
survey	20.00	1.93	14.24	10.98	11.20	14.77	1.82
survey+census	30.00	30.00	7.84	5.94	12.03	15.61	0.99
census	30.00	30.00	6.43	4.83	12.22	15.79	0.80
survey	30.00	1.93	14.24	10.98	11.20	14.77	1.82
survey+census	40.00	40.00	9.26	7.06	11.85	15.42	1.17
census	40.00	40.00	7.24	5.47	12.11	15.69	0.91
survey	40.00	1.93	14.24	10.98	11.20	14.77	1.82

Table S3. Accuracy metrics used to evaluate weigh optimization.

References

1. Maszk - hungarian data provider questionnaire, https://figshare.com/articles/online_resource/Hungarian_Data_Provider_Questionnaire/13550057.

2. Dr. Vilmos Bilicki MASZK Development Team, D. o. S. D., University of Szeged. Maszk app for android, https://play.google.com/store/apps/ (date of access 2020.10.02).
